# Differentiation, adaptation, and perseverance: Maturing conceptualizations of education-focused science faculty in the United States

**DOI:** 10.1371/journal.pone.0304426

**Published:** 2024-06-14

**Authors:** Seth D. Bush, Michael T. Stevens, Kimberly D. Tanner, Kathy S. Williams

**Affiliations:** 1 California State Polytechnic University, San Luis Obispo, San Luis Obispo, CA, United States of America; 2 Utah Valley University, Orem, UT, United States of America; 3 San Francisco State University, San Francisco, CA, United States of America; 4 San Diego State University, San Diego, CA, United States of America; The University of Sydney School of Biological Sciences: The University of Sydney School of Life and Environmental Sciences, AUSTRALIA

## Abstract

Science education reform has been underway for almost a century with the general aim to engage students and train scientists needed to find solutions to global challenges, and also ensure a general public well disposed towards science. In an effort to aid science reform, more recently, colleges and universities have been augmenting their academic workforce by embedding education-focused science faculty into science departments. However, little research has investigated how this approach, and the identity of these faculty, may be changing over time. Here we investigate how conceptualizations of professional identities of these faculty across the United States have changed over the last two decades. We found three professional identities amongst these faculty: Science Faculty with Education Specialties (SFES), Discipline-Based Education Researchers (DBER), and faculty who identify as *both* SFES and DBER. Evidence indicates this is a maturing field within higher-education science departments, with more direct hiring and training pathways, but with potentially diminishing agency. Finally, data reveal resilience and perseverance despite negative biases from peers and college administrators, especially at PhD-granting institutions.

## Introduction

Science education reform initiatives have received considerable attention in the past several decades, from increased funding to support STEM research and education after Sputnik [1], to a call to action to prepare undergraduates for 21st-century biology using research findings about effective teaching strategies reflecting current content and competencies [2–4]. However, much less attention has been focused on the faculty engaged in improving science education themselves. Understanding how the professional identity of these science education reformers interacts with these initiatives, especially over time, is essential. Initiatives in science education are more likely to achieve their aim if we understand the faculty who are doing the reforming.

Over nearly two decades, science departments in universities and colleges have embedded education-focused science faculty to focus on three arenas of science education including Science Education Research (SER) [5], K-12 Science Education (K-12) [4], and Undergraduate Science Education (UGSE) [6], and combinations of the three [7, 8]. These types of faculty have been studied in the United States, described as “Science Faculty with Educational Specialties” [9, 10 and references therein], and worldwide referred to as “higher education teaching staff” and “SoTL (Scholarship of Teaching and Learning) researchers” [11, 12], “teacher-researchers” and “Teaching Stream Faculty” in Canada [13, 14], “education/teaching focused academics” or “Teaching Specialists” in Australia [15–17], and “teaching-only academic staff” in the UK [18, 19]. This decades-long approach to improving science education provides an opportunity to look at science education reform longitudinally, and with a unique lens on higher-education faculty. This university-faculty focus contrasts with more typical longitudinal science education studies, which center on student achievement [20] or K-12 teachers [21–25].

The umbrella term Science Faculty with Education Specialties (SFES) (Table 1), introduced in 2006 [26] and first studied in 2008 [7], was used to describe education-focused science faculty who engaged in science education-related activities in the three arenas of science education: SER, K-12, and UGSE [7]. Studies of SFES have reported that these types of education-focused science faculty are poised to positively impact the way science education occurs within science departments but that the exact purpose of these positions is unclear [8]. In fact, the motivations for hiring SFES are sometimes misaligned with the contributions that SFES report making or aspire to make [27]. Nevertheless, SFES report influencing the teaching practice of their science department colleagues [28]. In the United States, SFES are widespread, increasing in numbers, and found at a variety of institution types, including primarily-undergraduate, MS-granting, and PhD-granting institutions [29], although the roles that they play at these institution types varies [30].

**Table 1:** Summary of key concepts.

**Faculty Identities Under Study**	
DBER	Discipline-Based Education Researchers
SFES	Science Faculty with Education Specialties
**Three Arenas of Science Education **	
SER	Science Education Research
K-12	K-12 Science Education
UGSE	Undergraduate Science Education

There are multiple examples of how these education-focused faculty are embedded in science departments across North America. Unlike SFES first described in 2008, who were hired with no difference in title from typical research faculty, SFES at other institutions may be in tenure-track positions that are parallel to others in their department but named differently than other disciplinary colleagues. The most notable example includes Professors of Teaching (PoTs), or Lectures with Potential for Security of Employment (LPSOE) and Lecturers with Security of Employment (LSOE)–aligned with Assistant and Associate Research Professors in the University of California system [31–34]. Another type of embedded educational change agents are primarily postdoctoral fellows, called Science Teaching and Learning Fellows (STLF) at the University of British Columbia in Canada [6].

Since the introduction of the term SFES by Bush et al. (2006) [26], evidence has suggested that the identities of education-focused science faculty are more nuanced and may be changing. Many faculty engaged as Discipline-Based Education Researchers (DBER) [35], originally envisioned as a subgroup of SFES, now view DBER as a distinct and different identity [10]. This could be related to the focus of DBER faculty on the role that research findings play in improving science education [36], whereas SFES tend to be engaged more broadly in multiple science-education arenas [7].

The overarching research objective for this study was to investigate how professional identities of education-focused science faculty in colleges and universities in the United States have changed over the last two decades. As such, we explore the identity of DBER, SFES, and a combination of *both* DBER and SFES. We provide evidence that education-focused science faculty have differentiated, adapted, and persisted over the past decades, even in the face of perceived negative biases noted previously [37]. This evidence supports the proposition that science education is maturing as a subdiscipline within the natural sciences.

## Materials and methods

### Survey development

Using insights from prior research studies [7, 10, 29] on SFES, we developed a 19-question, anonymous, online survey to investigate the academic positions, professional activities, characteristics, and experiences of education-focused science faculty in the United States (S1 Appendix). The majority of survey items were structured as multiple, closed-ended thematic prompts with opportunities to clarify answers through open-ended follow-ups. Operational definitions of terms used in Table 1 were included for clarity and consistency. In total, six items probed respondents’ professional contexts, three items probed professional identity, three items probed professional activity, three items probed professional experiences, and three items probed personal identity.

For most closed-ended items, respondents who believed they did not fit into one of the categories provided were given an opportunity to respond to an open-ended prompt to describe, “Something not listed here.” After reading through the range of open-ended responses for each prompt, researchers iteratively developed a coding strategy to identify and categorize responses. Responses were then independently coded to consensus by two members of the research team.

The survey was face validated by seven education-focused science faculty who represented a variety of academic positions, disciplines, and institution types. The study was approved by the Institutional Review Board (IRB) of California Polytechnic State University, San Luis Obispo.

### Sampling and data collection

Recruitment: Between March 1 and April 8, 2022, education-focused science faculty in the United States (n = 1,043) were contacted for this study via email using addresses they entered into a National Registry of Science Faculty with Education Specialties (SFES) in 2009 [29]. In addition, extensive announcements through email broadcasts and listservs were sent to professional societies in the sciences and multiple science education societies. Further, those who received invitations via the national registry or via listserv were encouraged to share our invitation with education-focused science faculty in their professional circles, effectively broadening our recruitment efforts. For direct invitees whose emails were returned, we searched for up-to-date email addresses using their names, disciplines, and institutions. One hundred sixty-five individuals were removed from our database because we could not find an updated email address for them, they had retired and left their campus, or they were no longer working in higher education, bringing our invitee list to 878 individuals. These individuals were sent up to three reminder emails encouraging them to participate and forward our invitation to their education-focused science faculty colleagues. Compensation was not offered to either the original invitees or to their referrals. A secure link at the end of the survey allowed participants to provide their contact information if they were interested in being contacted regarding a potential future follow-up interview study, while maintaining the anonymity of their survey responses.

Exclusion Criteria: After providing written informed consent to begin the online survey, following a procedure approved by the aforementioned IRB, 287 people responded to our survey. Of these 287 respondents, individuals who did not complete at least 50% of the survey were excluded (n = 27). Respondents whose positions were not in biology, chemistry, geosciences, physics, or other natural science departments were also excluded (n = 22). For example, we excluded respondents whose departments were outside the natural sciences, such as computer science, education, engineering, and mathematics departments. These two exclusion criteria brought our sample size for analysis to n = 238 respondents, of which 69 were hired before 2000, 115 were hired between 2000 and 2009, and 54 were hired after 2009.

### Statistical analysis

We used Pearson’s χ^2^ tests to assess whether paired observations were independent of each other (e.g., responses of DBER Only, Both DBER & SFES, or SFES Only; and start dates before 2000, 2000–2009, or after 2009). Each χ^2^ test reported in the Results section is the product of a different comparison between paired responses to survey questions. The specific measures are described with each reported test. A χ^2^ probability of 0.05 or less was used to justify rejecting the null hypothesis that the values from subpopulations of education-focused science faculty were unrelated to each other. Cramer’s V tests were used to estimate effect size (Φ_c_) for each Pearson’s χ^2^ test, Φ_c_ = 0.1–0.2 were considered modest correlations, Φ_c_ = 0.2–0.4 were considered moderate correlations, and Φ_c_ = 0.4–0.6 were considered relatively strong correlations. These ranges were based on the effect size classes of small Φ_c_ = 0.1, medium Φ_c_ = 0.3, and large Φ_c_ = 0.5 for crosstabulation data outlined by Cohen in 1988 [38].

Logistic models were developed to explore what factors were most predictive of reports of negative bias by education-focused science faculty. In building models, factors of identity (DBER, SFES, Hired specifically into an education-focused science faculty role, Transitioned into an education-focused science faculty role, and gender), status (tenure-track, non-tenure-track, hire date, and formal training), location (institution type, department/field), and scholarly work (publishing and/or writing grants in SER, K-12, or UGSE) were included. Models were refined and optimized by iteratively removing the least informative factors.

### Limitations

These findings describe education-focused science faculty in the United States, with representation from 35 states, Puerto Rico, and the District of Columbia. Participants reported being from a variety of institution types, the majority being from primarily-undergraduate institutions (PUIs), MS-granting institutions, or PhD-granting institutions with only nine from associate degree-granting institutions. Participants self-selected and were not compensated for their time. Recruitment utilized established listservs, professional societies, and peer-to-peer invitation. While this is not an exhaustive census of all education-focused science faculty in the United States, the parallels seen in this study and prior studies [10, 29] suggest that this pool is a reasonable proxy and that results presented here likely reflect the population of education-focused science faculty as a whole. With that said, we cannot rule out the possibility that our participant recruitment strategy may have biased our sample pool in ways we did not detect, which might reduce the generalizability of our results. In interpreting these results, it is important to note that of the 19 statistically significant findings presented, nine had modest correlations (Φ_c_ = 0.1–0.2) and ten had moderate correlations (Φ_c_ = 0.2–0.4), all ranging from effect size classes of small to medium based on Cohen [38].

## Results

### Differentiation: Conceptualizations of DBER and SFES

#### Three identities

Education-focused science faculty in the United States appear to have differentiated into three identities: ***DBER Only*** faculty, ***SFES Only***, and ***Both DBER & SFES*** (those with dual DBER and SFES identities). The phenomenon of education-focused faculty has changed over the last decade in multiple ways and also has shown some consistency. Adaptations were observed in training profiles, career pathways, and tenure-track status of education-focused science faculty and are described below. Perseverance was found in terms of demographics, negative bias, and the potential benefits of having education-focused peers [27]. PhD-granting institutions appeared to remain challenging environments for education-focused science faculty.

**Fig 1:**
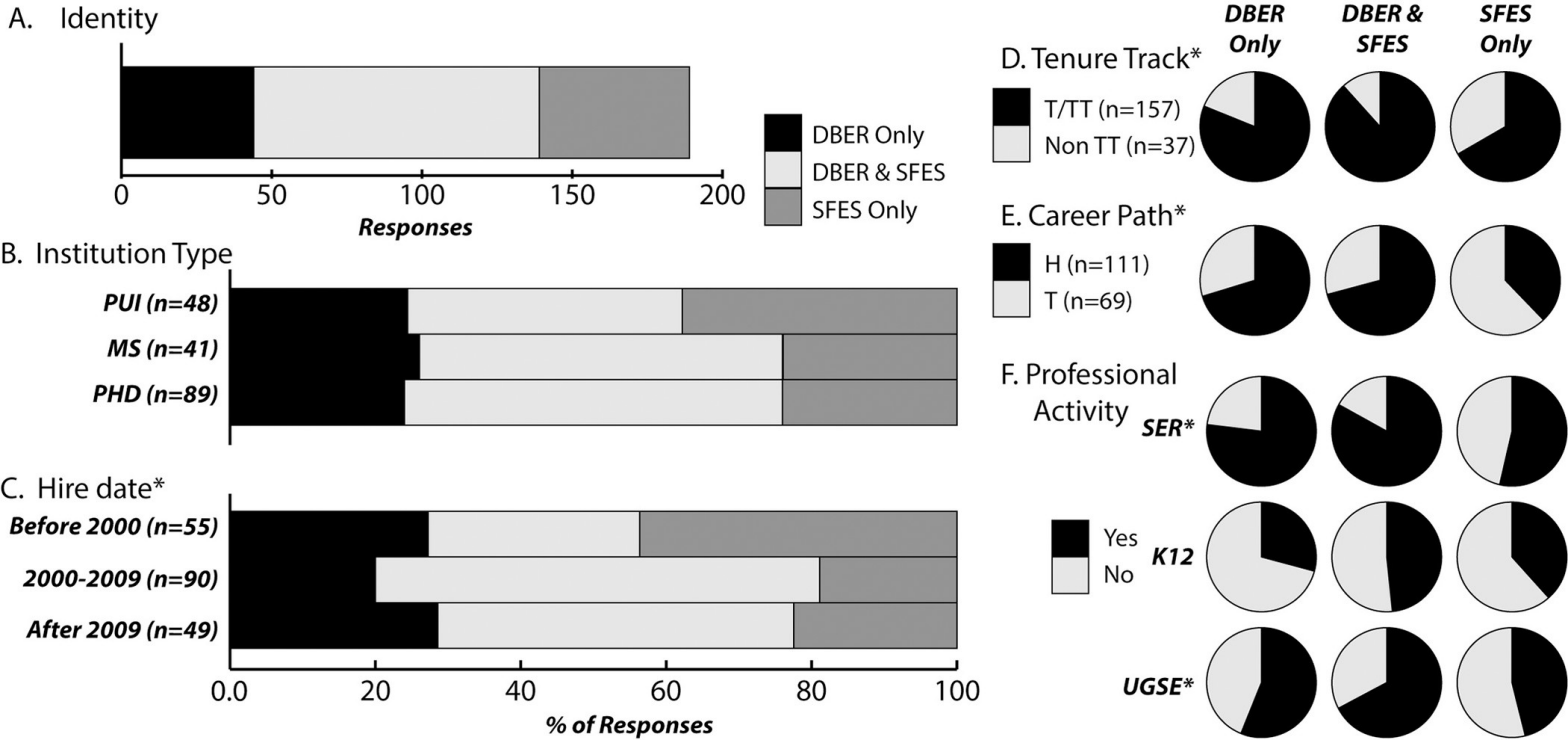
Differentiation in DBER and SFES conceptualizations. (**A**) Distribution of identities. (**B**) Identities across institution types. (**C**) Identities across hire-date ranges. *Those hired after 2000 were significantly more likely to report a DBER identity (DBER Only or Both DBER & SFES) than those hired before 2000 (χ^2^ = 9.92, df = 1, p = 0.002, Φ_c_ = 0.23). (**D**) Identities across position type (tenured/tenure-track [T/TT] vs. non-tenure-track [Non TT]). *Those with any DBER identity (DBER Only or Both DBER & SFES) were significantly more likely to be in a T/TT position than those who identified as SFES Only (χ^2^ = 7.91, df = 1, p = 0.005, Φ_c_ = 0.20). (**E**) Identities across career paths, hired specifically into an education-focused science faculty role (H) vs. transitioned into an education-focused science faculty role (T). *Those with a DBER Only or a Both DBER & SFES identity were significantly more likely to be hired specifically into an education-focused science faculty role than those who identified as SFES Only (DBER Only vs. SFES Only: χ^2^ = 8.64, df = 1, p = 0.003, Φ_c_ = 0.30; Both DBER & SFES vs. SFES Only: χ^2^ = 12.83, df = 1, p < 0.001, Φ_c_ = 0.31). (**F**) Identities across professional activity (applying for grants) in the three arenas of science education. *Those with any DBER identity (DBER Only or Both DBER & SFES) were significantly more likely to seek funding in science education research (SER) (81%, n = 143) or undergraduate science education (UGSE) (63%, n = 143) than those who identified as SFES Only (SER: 54% n = 52; UGSE: 46%, n = 52) (SER: χ^2^ = 13.31, df = 1, p < 0.001, Φ_c_ = 0.26; UGSE: χ^2^ = 4.12, df = 1, p = 0.042, Φ_c_ = 0.15).

In describing their professional identities, education-focused science faculty differentiated between DBER faculty and SFES. Most did not consider the terms DBER faculty and SFES to be equivalent (86.6%, n = 238). Of those who identified as DBER faculty and/or SFES, three identity categories were found (Fig 1A):

- DBER Only, those who identified as DBER faculty and not as SFES;
- SFES Only, those who identified as SFES and not as DBER faculty;
- Both DBER & SFES, those who identified as both a DBER faculty and as an SFES.

The largest proportion identified as Both DBER & SFES (40.8%, n = 239) (Fig 1A). Strikingly, even for individuals with this dual identity, the vast majority did not consider the terms DBER faculty and SFES to be equivalent terms (81.3%, n = 96).

#### Variation based on hire date, tenure-track status, and being hired specifically into an education-focused role

DBER/SFES identity distributions did not vary significantly across institution types (PUIs, MS-granting institutions, and PhD-granting institutions; Fig 1B). However, differences in identity distributions began to emerge upon exploring hiring pathways. For example, identity distributions differentiated significantly by hire date (Fig 1C). Those hired after 2000 were more likely to report any DBER identity (DBER Only or Both DBER & SFES) than those hired before 2000. Identity distributions also varied significantly by tenure-track status (Fig 1D). Those with any DBER identity (DBER Only or Both DBER & SFES) were more likely to be in tenured/tenure-track positions than those who identify as SFES Only. Finally, identity distributions significantly differentiated by career path: those hired specifically into an education-focused science faculty role (H) compared to those who transitioned into an education-focused science faculty role (T) (Fig 1E). Those with a DBER Only or a Both DBER & SFES identity were more likely to be hired specifically into an education-focused science faculty role than those who identified as SFES Only. Taken together, faculty with any DBER identity were more likely to have been hired more recently, into a tenure-track role, and specifically into an education-focused position.

#### Diverging professional activities

How education-focused science faculty engage in three arenas of science education (Science Education Research [SER], K-12 Science Education [K-12], and Undergraduate Science Education [UGSE]) varied by DBER/SFES identity. Focusing on reported solicitation of grant funding as one measure of professional activity within a given arena, SER is the highest reported activity and K-12 was the lowest reported activity across identities (Fig 1F). Those with Both DBER & SFES identity were more likely to engage in each of the three arenas. Education-focused science faculty who identified as a DBER faculty (either as DBER Only or as Both DBER & SFES) were significantly more likely to engage in SER or UGSE than those identifying as SFES Only. Similarly, dual identity faculty (Both DBER & SFES) reported engaging in publishing and mentoring activities more than DBER Only or SFES Only faculty (S1 Fig in S1 Appendix). Respondents with the dual identity were significantly more likely to report publishing in SER (94%, n = 95) or K-12 (42%, n = 95) than those identifying as DBER Only or SFES Only (SER: 69%, n = 100, K-12: 24% n = 100) (SER: χ^2^ = 17.74, df = 1, p < 0.001, Φ_c_ = 0.30; K-12: χ^2^ = 6.45, df = 1, p = 0.011, Φ_c_ = 0.18). Furthermore, respondents with the dual identity were significantly more likely to report mentoring a colleague in SER (72%, n = 95) or UGSE (76%, n = 95) than those with a DBER Only or SFES Only identity (SER: 43%, n = 100, UGSE: 60% n = 100) (SER: χ^2^ = 15.08, df = 1, p < 0.001, Φ_c_ = 0.28; UGSE: χ^2^ = 4.74, df = 1, p = 0.030, Φ_c_ = 0.16). Finally, those identifying as SFES Only were significantly more likely to mentor a colleague in UGSE than individuals with any DBER identity (i.e., DBER Only or Both DBER & SFES) (χ^2^ = 4.12, df = 1, p = 0.042, Φ_c_ = 0.15) (S1 Fig in S1 Appendix).

### Adaptation: Changes in the education-focused science faculty population in response to their environment

#### Increased formal training in science education

**Fig 2:**
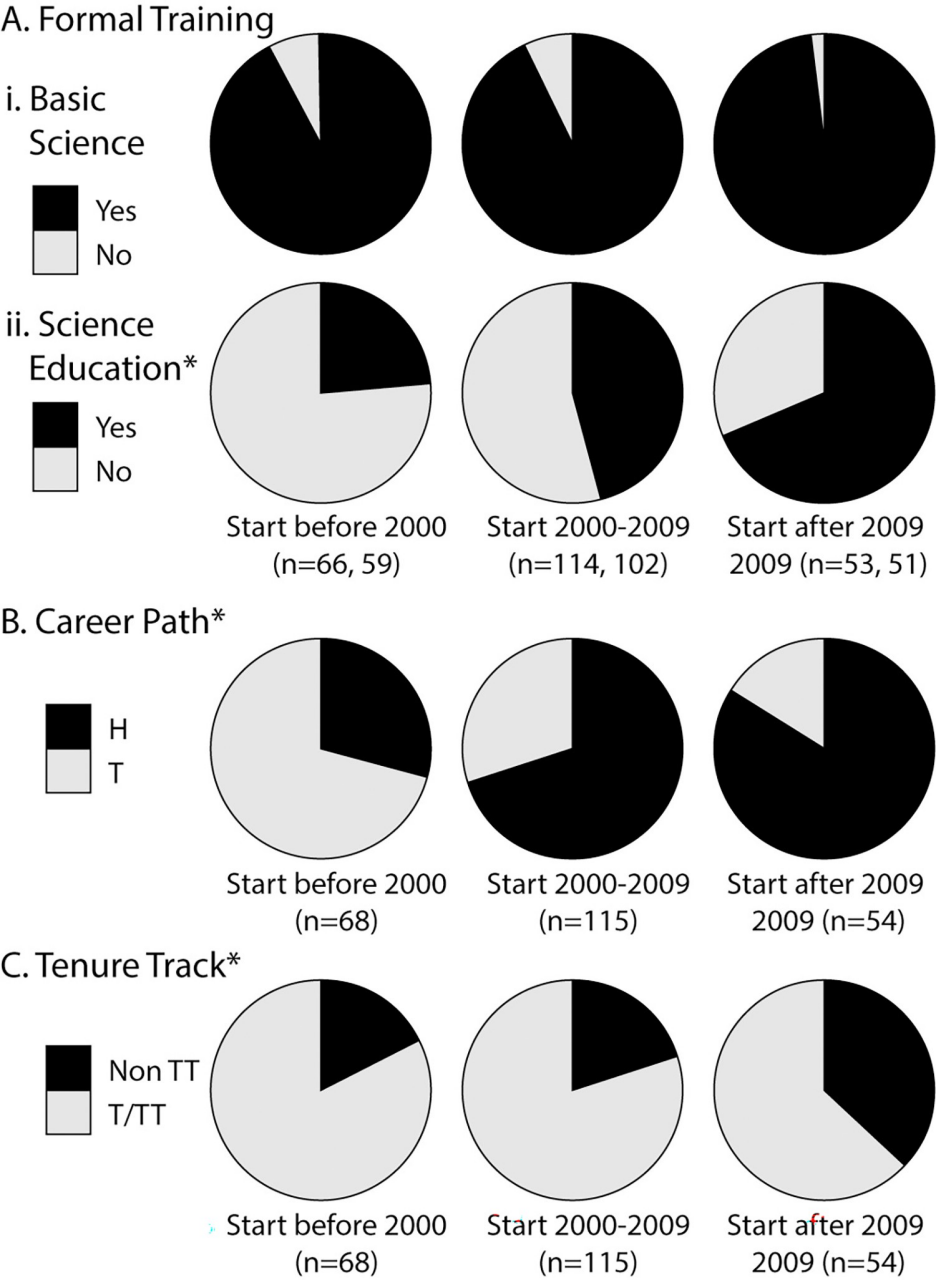
Adaptations over time. (**A**) Formal training in (**i**) basic science and (**ii**) science education disaggregated by hire date. Most education-focused science faculty had formal training in basic science. *Those starting after 2009 were the most likely to report formal science education training and those starting before 2000 were the least likely to report formal science education training (three-way comparison, χ^2^ = 4.86, df = 1, p = 0.003, Φ_c_ = 0.15; each group was significantly different from each other group). (**B**) Career path: those hired specifically into an education-focused science faculty role (H) and those who transitioned into an education-focused science faculty role (T), disaggregated by hire date. *Those starting after 2009 were the most likely to report being specifically hired into their education-focused positions and those starting before 2000 were the least likely to report being hired into their education-focused positions (three-way comparison, χ^2^ = 15.24, df = 1, p < 0.001, Φ_c_ = 0.28; each group was significantly different from each other group). (**C**) Tenure-track status disaggregated by hire date. *Those with start dates after 2009 were significantly more likely to report being in a non-tenure-track position (Non TT) vs. a tenured/tenure-track (T/TT) than those hired 2009 or before (χ^2^ = 6.53, df = 1, p = 0.011, Φ_c_ = 0.17).

Formal training in basic science or science education is defined here as postbaccalaureate training through a postdoctoral position and/or PhD or MS degree and/or, in the case of science education, a K-12 teaching credential or graduate fellowship in science education. Consistent with previous studies [10, 29], high (92%) percentages of education-focused science faculty reported training in basic science (Fig 2Ai). Percentages of training in basic science did not differ across DBER/SFES identity groups.

While percentages of science education training were lower than training in basic science (Fig 2Ai, 2Aii), they have increased significantly over time, growing from 24% for those with start dates before 2000, to 46% for those starting between 2000 and 2009, to 69% for those starting after 2009. Individuals with the dual identity (Both DBER & SFES) were significantly more likely to have formal training in science education (57%, n = 91) than faculty identifying as SFES Only or DBER Only (40%, n = 92) (χ^2^ = 4.39, df = 1, p = 0.032, Φ_c_ = 0.16).

#### More intentional hiring of education-focused science faculty

Career pathways for education-focused science faculty have become significantly more direct over time (Fig 2B). In earlier studies [7], two distinct career pathways were reported, including 1) faculty who were hired (H) specifically into their science education roles and 2) faculty who transitioned (T) into their science education roles after their initial hire with a specialty in another field (e.g., entomology). The population of education-focused science faculty specifically hired into their roles has grown significantly over time, from 29% for those with start dates before 2000, to 70% for those starting between 2000 and 2009, to 84% for those starting after 2009. As mentioned above (Fig 1E), those who identified as DBER Only or as Both DBER & SFES were significantly more likely to have been hired specifically into an education-focused science faculty role than those who identified as SFES Only.

#### Diminished tenure-track status

Even with more intentional hiring of education-focused science faculty, the likelihood of these positions being tenure track has decreased over time. In our sample, education-focused science faculty with start dates after 2009 were significantly less likely to report being in a tenure-track position (63%), than those hired in 2009 or before (81%) (Fig 2C). As mentioned above (Fig 1D), those with any DBER identity (DBER Only or Both DBER & SFES) were significantly more likely to be in a tenured/tenure-track position than those who identified as SFES Only.

### Perseverance: Consistency and persistence in spite of reported negative bias

**Fig 3:**
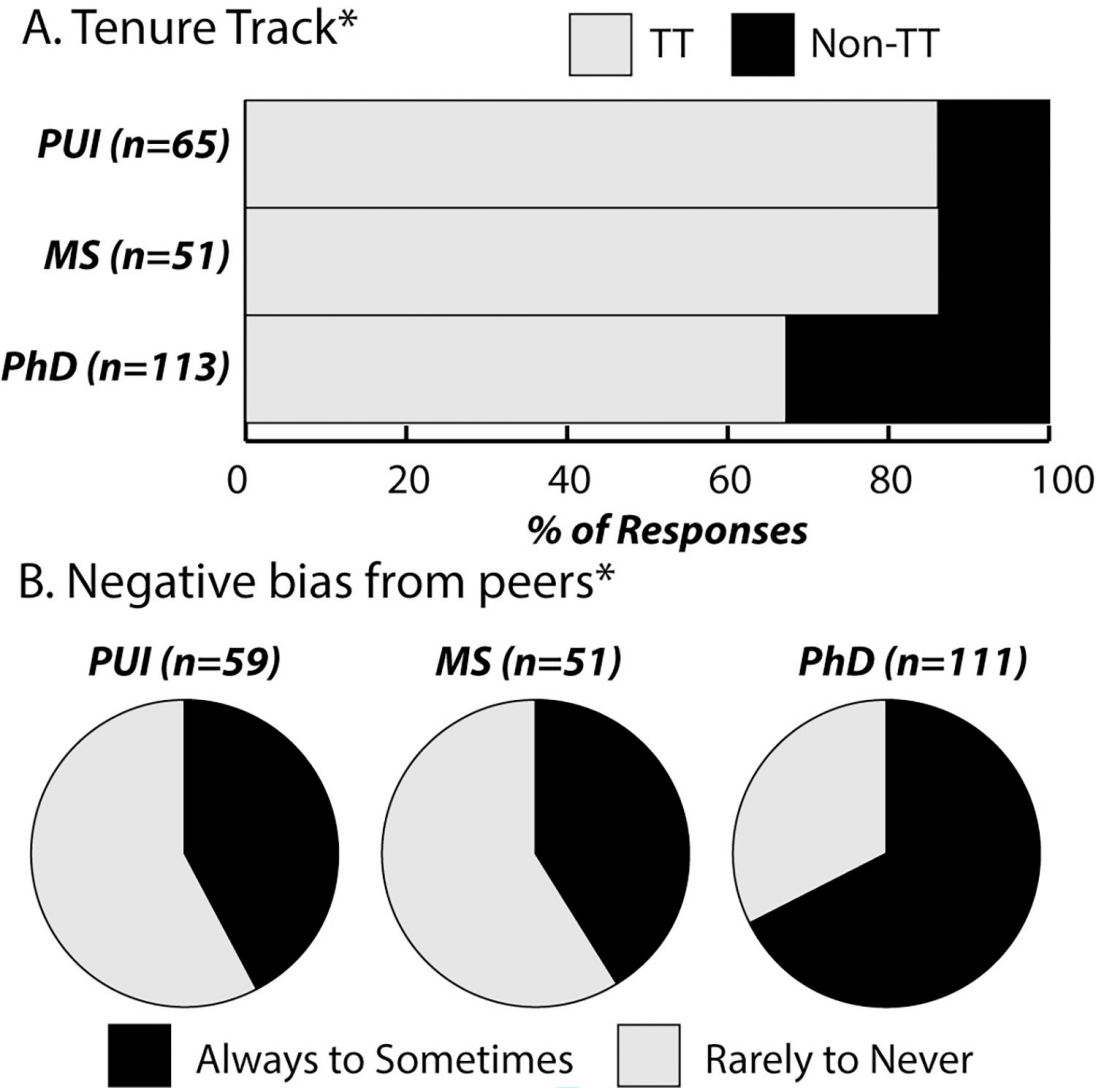
Perseverance across institution types. (**A**) Reports of negative bias from peers disaggregated by institution type. *Reports of bias from peers were significantly more pervasive at PhD-granting institutions (PhD) than at PUIs (χ^2^ = 9.08, d f = 1, p = 0.003, Φ_c_ = 0.19) or at MS-granting institutions (MS) (χ^2^ = 9.02, df = 1, p = 0.003, Φ_c_ = 0.18). (**B**) Tenure-track status disaggregated by institution type. *Faculty at PhD-granting institutions (PhD) were significantly less likely to be in a tenure-track position than faculty at PUIs (χ^2^ = 6.73, df = 1, p = 0.010, Φ_c_ = 0.23) or at MS-granting institutions (MS) (χ^2^ = 5.54, df = 1, p = 0.019, Φ_c_ = 0.24).

The phenomenon of education-focused science faculty has shown constancy in several ways. Distribution patterns across disciplines, institution types, gender identities (59% women), and racial identities (93% white) of education-focused faculty have remained consistent over time (S2 Fig in S1 Appendix) [10]. Additionally, these distribution patterns did not differ significantly across the DBER/SFES identities. Even with the recent decreases in tenure-track positions among education-focused science faculty (Fig 2C), the majority of these types of positions across all institution types are still tenured or tenure-track positions. That being said, education-focused science faculty at PhD-granting institutions continued to be less likely to be in a tenured or tenure-track position than faculty at PUIs or at MS-granting institutions (Fig 3A).

The tenacity of education-focused science faculty in somewhat fraught environments is evidenced by negative bias they report experiencing. A substantial portion reported that experiences of negative bias related to their work occurred "Always" to "Sometimes," and that negative bias by departmental peers was particularly acute at PhD-granting institutions (Fig 3B). Furthermore, reports of bias were proximal, significantly more likely be reported in relation to departmental peers (54%, n = 125), compared to university administrators (43%, n = 99) or members of the science community outside their home university (41%, n = 92), (p = 0.022 and p = 0.004, respectively). Despite perceived biases, the vast majority of respondents 94% (n = 220) report that they aspire to change how science is taught in their department or at their institution and 78% (n = 181) report they have been successful in this endeavor.

Logistic models were developed to explore what factor or factors (see Materials and Methods) were most predictive of reports of negative bias. Statistically-significant predictive models emerged for reports of faculty peer bias (S1 Table **Model 1** in S1 Appendix) and university administrator bias (S2 Table **Model 2** in S1 Appendix), however, no significant predictive model emerged for reports of bias from members of the science community beyond the home university.

When controlling for other factors, having a position at a PhD-granting institution was the most significant predictor of faculty peer bias. Those at PhD-granting institutions were 3.1 times more likely to report negative bias from peers than those at PUIs, and 3.8 times more likely to report negative bias from peers than those at MS-granting institutions.

Somewhat paradoxically, when controlling for other factors, the most significant predictor of reports of negative bias by university administrators related to scholarly activities, in particular publishing peer-reviewed articles related to science education. Those who reported publishing science education research were 3.8 times more likely to report they had experienced negative bias from administrators on their campus “Sometimes,” “Often,” or “Always” (Question 15 in S2 Appendix).

Having peers could be one factor that could contribute to the steadfastness of education-focused science faculty. Notwithstanding widespread bias against them, most education-focused science faculty reported having peers who specialize in science education in their department (56%) and/or college (82%; S3 Fig in S1 Appendix).

## Discussion

### Differentiation: A rise of a science-education research identity with associated bias

The complex stance among education-focused science faculty in the United States that DBER and SFES are non-equivalent, but sometimes overlapping, roles speaks to the complexity of science education as a field within the natural sciences [10]. Science education includes three arenas of activities: science education research, undergraduate science education, and K-12 science education. At many universities, research is more valued than teaching [6, 12, 33, 39–44]. It is quite possible that DBER faculty are benefiting from their orientation toward research and as a result are more likely to have been recently hired, to occupy a tenure-track or tenured position, and/or to have been specifically hired to attend to science education needs in their department. Although there is evidence for differentiation among education-focused science faculty, over 40% of the faculty in this study identified as Both DBER & SFES. Thus, as a group, education-focused science faculty have both changed and shown persistence over time, as described below.

The fact that education-focused science faculty in this study reported experiencing negative bias from college or university administrators is not without precedent. Biases like these have been directly documented in interview studies of College of Science deans [37]. What is novel and perplexing, however, is the correlation between negative bias and publishing peer-reviewed, science-education research articles. This seems to run counter to, and perhaps undermines, trends reported here toward education-focused science faculty adopting a DBER identity. Again, elements of this potential disconnect have been previously documented in interview studies of science deans [37] where ~30% of science deans held a negative impression of DBER faculty and 50% of deans reported that education-focused science faculty held a lower status than their science faculty peers on campus. Furthermore, seeds of this disconnect were seen in triangulated impact data [10]. While education-focused science faculty were more likely to report perceived impacts in the arena of science education research, deans were far less likely to value or acknowledge impacts in this arena and far more likely to recognize efforts in the arenas of undergraduate science education or K-12 science education. This disconnect represents differences in perspectives about what education-focused science faculty positions can contribute and may diminish the potential impact faculty in this role can have.

Within our logistic model, gender identity was not a statistically significant predictor of bias. However, the persistence of gender bias in the natural sciences may influence the experiences of education-focused science faculty in ways not detected with these analyses. For example, respondents in our study primarily identify as women and occupy education-focused academic roles, which could in turn result in these roles being conceptualized by some as academic “women’s work” with accompanying negative gender bias [16].

### Adaptation: A maturing field with potentially diminishing agency

The increased formal training in science education and more intentional hiring of education-focused science faculty both indicate a maturing field and, perhaps, a normalization of the education-focused science faculty phenomenon. The development of a new specialty often brings with it the requirement of new skills, training expectations, and formalized training pathways to delimit it from other fields [45]. Our study shows that science departments from across the United States are recognizing the value of education-focused faculty and seeking to add a new discipline to their programs.

At the same time that interest in hiring education-focused science faculty is increasing, there has been a reduction in the proportion of these positions that are tenure track, especially at PhD-granting institutions. This finding may very well echo the decline of tenure-track positions in general [18, 19, 46–49], perhaps exacerbated by the negative bias often held against education-focused science faculty [37, and reported here]. The degree to which occupying a non-tenure-track position may bring a decrease in agency among more recently hired education-focused science faculty and the impact this might have on their work and career trajectories is not clear. However, to flourish like other disciplines, science education may need tenure-track or tenured advocates who have the status and job security to lobby stridently for resources within their department, college, and institution.

### Perseverance: A decade of resilience and community with challenges, particularly at research-intensive institutions

The fact that demographic patterns among education-focused science faculty have been similar for over a decade [7, 10] further supports the institutionalization of this type of faculty. This could imply that as education-focused science faculty retire, they are being replaced in-kind by their departments. Although it is unfortunate that the body of science faculty is not becoming more racially diverse over time [50, 51], the majority of education-focused science faculty in this study reported being women, despite continuing underrepresentation of women among STEM faculty [52].

The negative bias that education-focused science faculty report experiencing from their peers, administrators, and the science community was triangulated by Bush et al. [37] who documented that College of Science deans also reported education-focused science faculty being discriminated against, also see Ross et al. [17]. In a culture that values scholarship over other endeavors, having a community of faculty focused on science education can have positive effects [53]. In this study we found that the majority of education-focused science faculty had peers who specialize in science education in their department and/or college, which might mitigate those biases (S3 Fig in S1 Appendix).

The negative bias against education-focused science faculty appears most prevalent at PhD-granting institutions. At these research-intensive universities, teaching (and those who focus on teaching) can be relegated to the margins [39–41]. A manifestation of this was the lower percentages of education-focused science faculty who hold tenured or tenure-track positions at PhD-granting institutions, as opposed to PUIs or MS-granting institutions.

## Conclusion

Prior to this study, little research has focused on the professional identities of faculty engaged in improving science education, particularly over time. Evidence presented here addresses this important gap in the literature. Here we report that science education has matured as a subdiscipline within the natural sciences over the last two decades. This is evidenced by education-focused science faculty: 1) encompassing multiple professional identities including DBER, SFES, and their combination, 2) having more formal training in science education, and 3) being more likely to be hired into positions intentionally crafted for science education specialists. Despite the maturation of the field, education-focused science faculty, especially those with research identities, continue to contend with negative biases held by their peers and college administrators. Additionally, tenure-track positions are becoming less common. These challenges could hamper the science education reform efforts of both DBER faculty and SFES, especially at PhD-granting universities.

## Supporting information

S1 Appendix Supporting information with S1-S3 Figs and S1, S2 Tables. (PDF)

S2 Appendix SFES survey instrument. (PDF)

## References

[1] Brainard J. 50 years after Sputnik, America sees itself in another science race. Chron High Educ. 2007 Oct 12;54(7):A22–3.

[2] Brewer CA, Smith D. Vision and change in undergraduate biology education: a call to action. American Association for the Advancement of Science, Washington, DC. 2011 Feb;81.

[3] Olson S, Riordan DG. Engage to excel: producing one million additional college graduates with degrees in science, technology, engineering, and mathematics. Report to the president. Executive Office of the President. 2012 Feb.

[4] National Research Council. Next generation science standards: For states, by states. 2013.

[5] National Research Council, Singer SR, Nielsen NR, Schweingruber HA. Discipline-based education research: Understanding and improving learning in undergraduate science and engineering. Washington, DC: National Academies Press; 2012.

[6] Code WJ, Welsh AJ, Maxwell EJ. A longitudinal perspective of the experiences and career trajectories of discipline-based education specialists in teaching and learning in higher education. Int J Acad Dev. 2023 May 26:1–4.

[7] Bush SD, Pelaez NJ, Rudd JA, Stevens MT, Tanner KD, Williams KS. Science faculty with education specialties. Science. 2008 Dec 19;322(5909):1795–6. 19095927 10.1126/science.1162072

[8] Bush SD, Pelaez NJ, Rudd JA, Stevens MT, Tanner KD, Williams KS. Investigation of science faculty with education specialties within the largest university system in the United States. CBE Life Sci Educ. 2011 Mar 1;10(1):25–42. doi: 10.1187/cbe.10-08-0106 21364098 PMC3046885

[9] Addy TM, Simmons P, Gardner GE, Albert J. A new" class" of undergraduate professors: Examining teaching beliefs and practices of science faculty with education specialties. J Coll Sci Teach. 2015 Jan 1;44(3):91–9.

[10] Bush SD, Stevens MT, Tanner KD, Williams KS. Evolving roles of scientists as change agents in science education over a decade: SFES roles beyond discipline-based education research. Sci Adv. 2019 Jun 5;5(6):eaav6403. 10.1126/sciadv.aav6403 PMC6551186 31183399

[11] Chevaillier T. The changing conditions of higher education teaching personnel. Geneva: International Labour Office; 2000 Jul.

[12] Billot J, Rowland S, Carnell B, Amundsen C, Evans T. How experienced SoTL researchers develop the credibility of their work. Teac. Learn Inq. 2017 Mar 29;5(1):101–14.

[13] Clark ID, Moran G, Skolnik ML, Trick D. Academic transformation: The forces reshaping higher education in Ontario. Kingston, ON: McGill-Queen’s University Press; 2009.

[14] Hubball HT, Burt H. The scholarship of teaching and learning: Theory–practice integration in a faculty certificate program. Innov Higher Educ. 2006 Mar;30:327–44.

[15] Ross PM. The changing nature of the academic role in Science. Canberra: Department of Education; 2019. Available from: https://ltr.edu.au/resources/FS14-0232_Ross_FinalReport_2019.pdf.

[16] Ross PM. Women’s work: Education-focused academics in higher education. In Women thriving in academia 2021 Apr 26 (pp. 109–128). Emerald Publishing Limited.

[17] Ross PM, Scanes E, Poronnik P, Coates H, Locke W. Understanding STEM academics’ responses and resilience to educational reform of academic roles in higher education. International journal of STEM education. 2022 Jan 28;9(1):11. doi: 10.1186/s40594-022-00327-1 35127335 PMC8796749

[18] Wolf A, Jenkins A. Why have universities transformed their staffing practices? An investigation of changing resource allocation and priorities in higher education. Social Research Institute, Kings College London and the University College London; 2020 Dec. Available from: https://discovery.ucl.ac.uk/id/eprint/10118762/1/why-have-universities-transformed-their-staffing-practices.pdf.

[19] Wolf A, Jenkins A. Managers and academics in a centralising sector: The new staffing patterns of UK Higher Education. The Policy Institute, King’s College London (KCL): London, UK; 2021 Dec. Available from: https://www.kcl.ac.uk/policy-institute/assets/managers-and-academics-in-a-centralising-sector.pdf.

[20] Le V-N, Stecher BM, Lockwood JR, Hamilton LS, Robyn A. Improving mathematics and science education: A longitudinal investigation of the relationship between reform-oriented instruction and student achievement. Rand Corporation; 2006 Sep 25.

[21] Forbes CT, Davis EA. Beginning elementary teachers’ learning through the use of science curriculum materials: A longitudinal study. In Annual meeting of the National Association for Research in Science Teaching, April, New Orleans. Available from: http://hice org/presentations/documents/Forbes_Davis_NARST2007.pdf; 2007 Apr.

[22] Fletcher SS, Luft JA. Early career secondary science teachers: A longitudinal study of beliefs in relation to field experiences. Sci Educ. 2011 Nov;95(6):1124–46.

[23] Ambusaidi AK, Al-Balushi SM. A Longitudinal Study to Identify Prospective Science Teachers’ Beliefs about Science Teaching Using the Draw-a-Science-Teacher-Test Checklist. Int J Environ Sci Educ. 2012 Apr;7(2):291–311.

[24] McKinnon M, Lamberts R. Influencing science teaching self-efficacy beliefs of primary school teachers: A longitudinal case study. Int J Sci Educ, Part B. 2014 Apr 3;4(2):172–94.

[25] Buldur S. A longitudinal investigation of the preservice science teachers’ beliefs about science teaching during a science teacher training programme. Int J Sci Educ. 2017 Jan 2;39(1):1–19.

[26] Bush SD, Pelaez NJ, Rudd JA, Stevens MT, Williams KS, Allen DE, et al. On hiring science faculty with education specialties for your science (not education) department. CBE Life Sci Educ. 2006 Dec;5(4):297–305. doi: 10.1187/cbe.06-09-0189 17146032 PMC1681366

[27] Bush SD, Pelaez NJ, Rudd JA, Stevens MT, Tanner KD, Williams KS. Misalignments: Challenges in cultivating science faculty with education specialties in your department. BioScience. 2015 Jan 1;65(1):81–9.

[28] Bush SD, Rudd JA, Stevens MT, Tanner KD, Williams KS. Fostering change from within: Influencing teaching practices of departmental colleagues by science faculty with education specialties. PLoS One. 2016 Mar 8;11(3):e0150914. doi: 10.1371/journal.pone.0150914 26954776 PMC4783031

[29] Bush SD, Pelaez NJ, Rudd JA, Stevens MT, Tanner KD, Williams KS. Widespread distribution and unexpected variation among science faculty with education specialties (SFES) across the United States. Proc Natl Acad Sci U S A. 2013 Apr 30;110(18):7170–5. doi: 10.1073/pnas.1218821110 23589844 PMC3645507

[30] Bush SD, Stevens MT, Tanner KD, Williams KS. Origins of science faculty with education specialties: Hiring motivations and prior connections explain institutional differences in the SFES phenomenon. BioScience. 2017 May 1;67(5):452–63.

[31] University of California Office of the President. Appointment and Promotion, Lecturer with Security of Employment Series (APM–285). 2018 October 10. Available from: https://www.ucop.edu/academic-personnel-programs/_files/apm/apm-285.pdf.

[32] Harlow A, Lo SM, Saichaie K, Sato BK. Characterizing the University of California’s tenure-track teaching position from the faculty and administrator perspectives. PLoS One. 2020 Jan 13;15(1):e0227633. doi: 10.1371/journal.pone.0227633 31929599 PMC6957150

[33] Harlow AN, Buswell NT, Lo SM, Sato BK. Stakeholder perspectives on hiring teaching-focused faculty at research-intensive universities. International J STEM Educ. 2022 Aug 9;9(1):54.

[34] Paine AR, Wilton M, Solanki SM, Ellefson M, Ferguson JE, Lo SM, et al. Reimagining the Role of Teaching-Focused Faculty in Research-Intensive Universities: The Evolution of Scholarly Expectations and Departmental Influence. 2024 Jan. Available from: https://bpb-us-w2.wpmucdn.com/wp.ovptl.uci.edu/dist/6/18/files/2024/01/Reimagining-the-Role-of-Teaching-Focused-Faculty-in-Research-Intensive-Universities-File-Leanna-Fong-2eef98f7a7d61aa5.pdf 10.1371/journal.pone.0334895 PMC12530556 41100536

[35] National Research Council. Discipline-based education research: Understanding and improving learning in undergraduate science and engineering. National Academies Press; 2012 Aug 27.

[36] Talanquer V. DBER and STEM education reform: Are we up to the challenge?. Journal of Research in Science Teaching. 2014 Aug;51(6):809–19.

[37] Bush SD, Stevens MT, Tanner KD, Williams KS. Disciplinary bias, money matters, and persistence: Deans’ perspectives on science faculty with education specialties (SFES). CBE Life Sci Educ. 2020;19(3):ar34. doi: 10.1187/cbe.19-10-0202 32762598 PMC8711804

[38] Cohen J. Statistical power analysis for the behavioral sciences. 2nd ed. Hillsdale: Lawrence Erlbaum; 1988.

[39] Serow RC. Research and teaching at a research university. High Educ. 2000 Dec;40:449–63.

[40] Fairweather J. Linking evidence and promising practices in science, technology, engineering, and mathematics (STEM) undergraduate education. Board of Science Education, National Research Council, The National Academies, Washington, DC. 2008 Oct 13.

[41] Fairweather JS. Beyond the rhetoric: Trends in the relative value of teaching and research in faculty salaries. J Higher Educ. 2005 Jul 1;76(4):401–22.

[42] Macfarlane B. Prizes, pedagogic research and teaching professors: lowering the status of teaching and learning through bifurcation. Teach High Educ. 2011 Feb 1;16(1):127–30.

[43] Macfarlane B. The morphing of academic practice: Unbundling and the rise of the para‐academic. High Educ Q. 2011 Jan;65(1):59–73.

[44] Ross PM, Scanes E, Locke W. Stress adaptation and resilience of academics in higher education. Asia Pacific Education Review. 2023 Feb 22:1–21.

[45] Gieryn TF. Boundary-work and the demarcation of science from non-science: Strains and interests in professional ideologies of scientists. Am Sociol Rev. 1983 Dec 1:781–95.

[46] Finkelstein MJ, Schuster JH. Assessing the silent revolution how changing demographics are reshaping the academic profession. AAHE Bull. 2001;54(2):3–7.

[47] Dobbie D, Robinson I. Reorganizing higher education in the United States and Canada: The erosion of tenure and the unionization of contingent faculty. Labor Stud J. 2008 Jun;33(2):117–40.

[48] Colby G. Data Snapshot: Tenure and Contingency in US Higher Education. American Association of University Professors. Academe. 2023 Spring 109(2). Available from: https://www.aaup.org/sites/default/files/AAUP%20Data%20Snapshot.pdf

[49] Stein M. The end of faculty tenure and the transformation of higher education. The end of faculty tenure and the transformation of higher education. Academe. 2023 Winter; 109(1). Available from: https://www.aaup.org/article/end-faculty-tenure-and-transformation-higher-education#.Y-PboXbMJPY

[50] Valantine HA, Lund PK, Gammie AE. From the NIH: A systems approach to increasing the diversity of the biomedical research workforce. CBE Life Sci Educ. 2016 Sep;15(3):fe4. doi: 10.1187/cbe.16-03-0138 27587850 PMC5008902

[51] Harris M, Rosser S, Goldman M, Márquez-Magaña L, Rohlfs RV. Improving biology faculty diversity through a co-hiring policy and faculty agents of change. PLoS One. 2023 May 15;18(5):e0285602. doi: 10.1371/journal.pone.0285602 37186580 PMC10184900

[52] Wapman KH, Zhang S, Clauset A, Larremore DB. Quantifying hierarchy and dynamics in US faculty hiring and retention. Nature. 2022 Oct 6;610(7930):120–7. doi: 10.1038/s41586-022-05222-x 36131023 PMC9534765

[53] Roxå T, Mårtensson K. Significant conversations and significant networks–exploring the backstage of the teaching arena. Stud High Educ. 2009 Aug 1;34(5):547–59.

