# Supplementary material for: Differentiation, adaptation, and perseverance: Maturing conceptualizations of education-focused science faculty in the United States
Source: PLoS One. 2024 Jun 14;19(6):e0304426. doi: 10.1371/journal.pone.0304426 (PMC11178152; doi:10.1371/journal.pone.0304426)
Supplement: S1 Appendix — (PDF) [file pone.0304426.s001.pdf]

Supporting Information for

Differentiation, adaptation, and perseverance: Maturing conceptualizations of education-focused science faculty in the United States

Seth D. Bush, Michael T. Stevens, Kimberly D. Tanner, Kathy S. Williams*

*Corresponding author. Email: kathy.williams@sdsu.edu

This PDF file includes S1 Appendix:

S1 Fig 1. Comparisons of professional activities in SER, K-12 Education, and UGSE disaggregated by DBER/SFES identity.
S1 Fig 2. Distributions of education-focused science faculty.
S1 Fig 3. Reports of science education-focused peers disaggregated by DBER/SFES identity.
S1 Table 1. Model 1.
S1 Table 2. Model 2.

S1 Fig 1

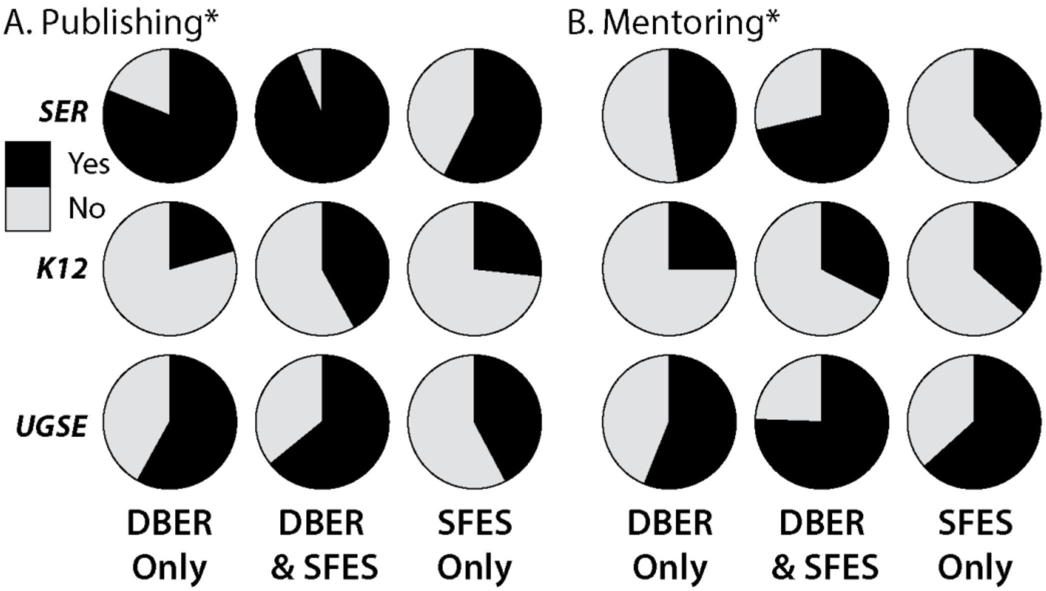

S1 Fig 1. Comparisons of professional activities in SER, K-12 Education and UGSE disaggregated by DBER/SFES identity. (**A**) Publishing peer-reviewed manuscripts. *Respondents with a dual identity (Both DBER & SFES) are significantly more likely to report publishing in SER (94%, n = 95) or K-12 (42%, n = 95) than those who had a DBER Only or SFES Only identity (SER: 69%, n = 100, K-12: 24%, n = 100) (SER: $\chi 2 = 17.74$, df = 1, $p < 0.001$; K-12: $\chi 2 = 6.45$, df = 1, p = 0.011). (**B**) Mentoring colleagues. *Respondents with a dual identity (Both DBER & SFES) were significantly more likely to report mentoring a colleague in SER (72%, n = 95) or UGSE (76%, n = 95) than those identifying as DBER Only or SFES Only (SER: 43%, n = 100, UGSE: 60%, n = 100) (SER: $\chi 2 = 15.08$, df = 1, $p < 0.001$; UGSE: $\chi 2 = 4.74$, df = 1, p = 0.030).

S1 Fig 2

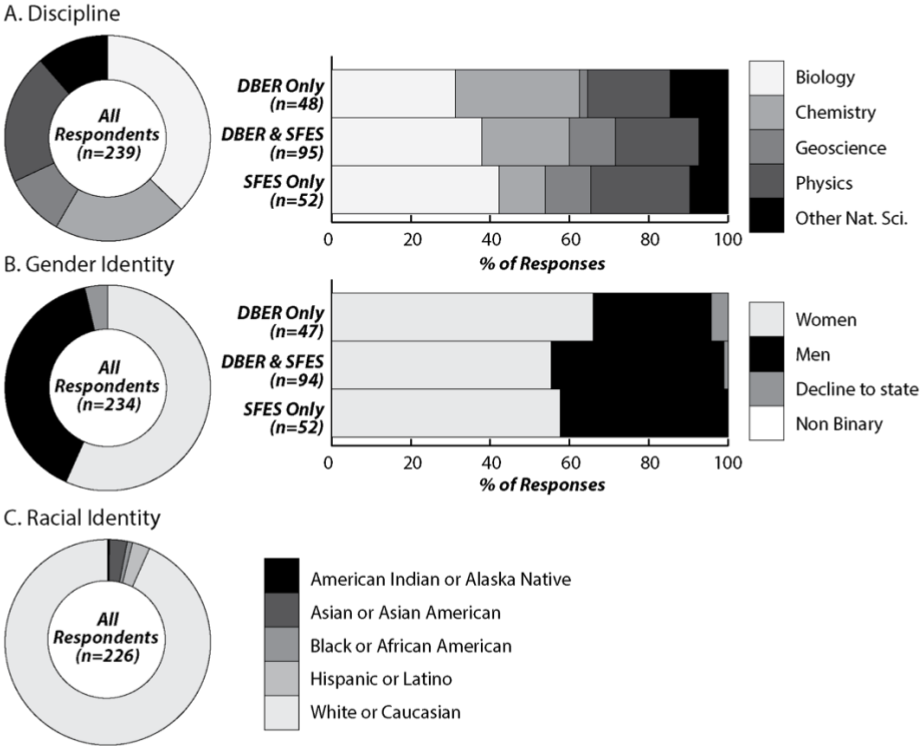

S1 Fig 2. Distributions of education-focused science faculty. (**A**) Discipline disaggregated by DBER/SFES identity, (**B**) Gender identity disaggregated by DBER/SFES identity, and (**C**) Racial identity.

S1 Fig 3

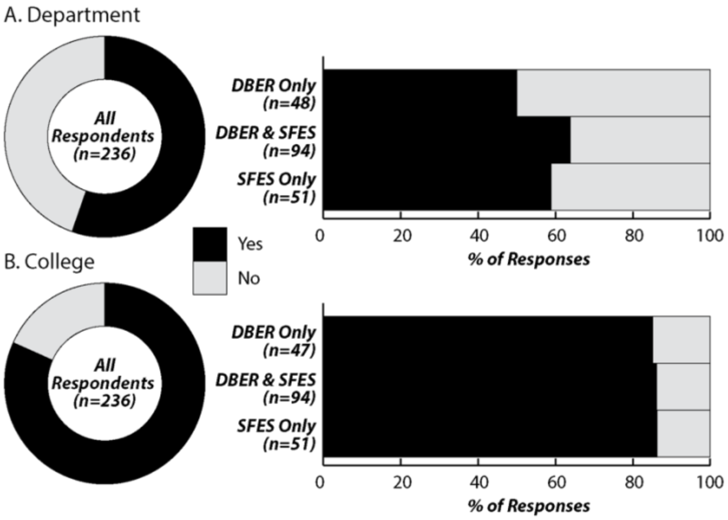

S1 Fig 3. Reports of science education-focused peers disaggregated by DBER/SFES identity. (**A)** Reported peers in home department and (**B**) Reported peers in college.

S1 Table 1

S1 Table 1. Model 1. Reports of negative bias by departmental peers more than rarely, binary logistical parameter estimates.

Term	Odds Ratio	Estimate	Std Error	$\chi2$	p-value
Intercept		0.0577	0.1610	0.13	0.720
Institution Type [MS]	0.8173	-0.5147	0.2482	4.3	0.038*
Institution Type [PHD]	3.1291	0.8277	0.2112	15.36	<.0001*
Formal Training Science Education [No]	0.5556	-0.2938	0.1582	3.45	0.063
Applied for grants K-12 [No]	0.6424	-0.221	0.1628	1.85	0.174
Applied for grants UGSE [No]	0.6035	-0.2525	0.1594	2.51	0.113

Whole model test $\chi2 = 25.08$; $p < 0.001$; Total population = 230 (AA (n = 9) excluded from analysis), negative bias by peers rarely to never: n = 105, negative bias by peers more than rarely n = 125. *$p < 0.05$

S1 Table 2

S1 Table 2. Model 2. Reports of negative bias by administrators more than rarely, binary logistical parameter estimates.

Term	Odds Ratio	Estimate	Std Error	$\chi2$	p-value
Intercept		-0.9421	0.3610	6.81	0.009*
Hired or Transitioned [Hired]	1.4017	-0.1689	0.1757	0.92	0.337
Formal Training In Science [No]	2.0843	-0.3672	0.3129	1.38	0.241
Published SER [No]	3.7531	-0.6613	0.2472	7.16	0.008*

Whole model test $\chi2$ = 9.739; p = 0.021; Total population = 230 (AA (n = 9) excluded from analysis, HT mix (n = 9) excluded from analysis), negative bias by peers rarely to never: n = 116, negative bias by peers more than rarely n =9 5. *p < 0.05
