# Supplementary material for: Differentiation, adaptation, and perseverance: Maturing conceptualizations of education-focused science faculty in the United States
Source: PLoS One. 2024 Jun 14;19(6):e0304426. doi: 10.1371/journal.pone.0304426 (PMC11178152; doi:10.1371/journal.pone.0304426)
Supplement: S2 Appendix — (PDF) [file pone.0304426.s002.pdf]

Supporting Information for

Differentiation, adaptation, and perseverance: Maturing conceptualizations of education-focused science faculty in the United States

Seth D. Bush, Michael T. Stevens, Kimberly D. Tanner, Kathy S. Williams*

*Corresponding author. Email: kathy.williams@sdsu.edu

This PDF file includes:

S2 Appendix. SFES survey instrument

Informed Consent

Dear Colleague,

You are being asked to participate in a research study investigating the experiences of education-focused science faculty on campuses across the United States. This study is being conducted by a research team of science faculty: Dr. Seth Bush at Cal Poly San Luis Obispo, Dr. Michael Stevens at Utah Valley University, Dr. Kimberly Tanner at San Francisco State University, and Dr. Kathy Williams at San Diego State University.

PURPOSE OF THE STUDY: The purpose of this study is to investigate the positions, characteristics, and experiences of education-focused science faculty.

PROCEDURES: You will be asked to complete an online survey that asks you questions about your current professional position and professional activities, as well as your perceptions about issues related to your position. If you volunteer to participate in this study, you will complete the survey anonymously using a secure website. You will be giving us permission to read, analyze, and report data resulting from your anonymous survey responses. The survey should take approximately 10 minutes to complete. Within this survey you will be asked if you are interested in participating in a potential future interview study. To maintain confidentiality, you will be given a link to a follow-up interest survey where, if you choose to do so, you can share your contact information.

POTENTIAL RISKS AND DISCOMFORTS: You are unlikely to be exposed to any potential risks or discomforts by participating in this study.

POTENTIAL BENEFITS TO YOU AND/OR SOCIETY: While there likely will not be an immediate direct benefit to you as a result of your participation in this study, higher education will potentially benefit from an investigation of the experiences of this group of faculty. University administrators, faculty candidates, and faculty hires will have data that may enhance hiring and retention for this type of faculty position.

PAYMENT FOR PARTICIPATION: You will not be paid for participating in this research project.

CONFIDENTIALITY AND ANONYMITY: Your identity will be kept strictly confidential during the entire research process, and the survey data itself will be collected anonymously. It may be possible for the researchers analyzing data from this survey to identify participants due to the amount of information about institution, department, faculty position, demographics, etc. We are interested in the set of responses as a whole, not a particular individual's responses. Confidential data or findings from this study might be included in various publications or presentations. Electronic survey data will be retained on a secure electronic platform for a minimum of 5 years.

PARTICIPATION AND WITHDRAWAL: You can choose whether to participate, and you may withdraw from the study at any time. Choosing not to participate or choosing to withdraw at any point will mean that your responses will not be included in data analysis or reporting for research purposes. You also do not have to answer any questions you choose not to.

IDENTIFICATION OF INVESTIGATORS: If you have any questions or concerns about the study, please feel free to contact Dr. Bush, via phone: (805) 756-2746 or email: sbush@calpoly.edu.

RIGHTS OF RESEARCH PARTICIPANTS: You may withdraw your consent at any time and discontinue participation without penalty. If you have concerns regarding the manner in which the study is conducted, you may contact Dr. Michael Black, Chair of the Cal Poly Institutional Review Board, at (805) 756-2894, mblack@calpoly.edu, or Ms. Trish Brock, Director of Research Compliance, at (805) 756-1450, pbrock@calpoly.edu.

* 1. I have read the procedures described above. By checking "Agree" below, I am electronically signing this document and consenting to participate in this study.

◯ Agree

◯ Disagree

2022 Perspectives of Education-focused Science Faculty in the United States

About you and your focus on science education...

Education-focused science faculty in the United States have a wide range of professional identities, interests, and training. Please use the lens of your current academic position to address each of the following prompts. While you may have some familiarity with one or both of the terms "DBER" and "SFES," please carefully read the definitions offered below and respond based on these definitions.

DBER (discipline-based education research) faculty are individuals with specialized and advanced expertise in a STEM discipline who conduct education research about the concepts, practices, culture, and ways of thinking of that discipline.

SFES (science faculty with education specialties) are faculty in science departments who focus on issues in science education beyond typical faculty teaching duties.

2. Based on the definition above, do you consider yourself to be a **Discipline Based Science Education Research (DBER) Faculty**?

- ◯ Yes, I was **hired** as a DBER faculty
- ◯ Yes, I **transitioned** into a DBER faculty role after my initial hire
- ◯ No, I engage in science education scholarship but this definition does not fit me
- ◯ No, I do not engage in DBER beyond typical faculty teaching duties
- ◯ No, I am not a faculty member in a science department
- ◯ I'm not sure because ...

3. Based on the definition above, do you consider yourself to be a **Science Faculty with an Education Specialty (SFES)**?

- ◯ Yes, I was **hired** as an SFES
- ◯ Yes, I **transitioned** into an SFES role after my initial hire
- ◯ No, I engage in science education scholarship but this definition does not fit me
- ◯ No, I do not engage in science education beyond typical faculty teaching duties
- ◯ No, I am not a faculty member in a science department
- ◯ I'm not sure because ...

4. Please consider each prompt.

	Yes	No
Do you identify as a DBER faculty?	○	○
Do you identify as an SFES?	○	○
Do you consider the terms DBER faculty and SFES to be equivalent to one another?	○	○
Do you aspire to be a change agent in your department or institution?	○	○
Do you aspire to be a change agent beyond your institution?	○	○

2022 Perspectives of Education-focused Science Faculty in the United States

About you and your position...

5. When were you hired into your position?

- before 1960
- 1960-1969
- 1970-1979
- 1980-1989
- 1990-1999
- 2000-2009
- 2010-2019
- 2020 or after

6. Which best describes your tenure status?

- I am Tenured
- I am in a Tenure-Track position, but am currently not Tenured
- I am in a Non-Tenure-Track position
- Something not listed here

7. Which field designation best reflects the Science Department that houses your position?

- Biology
- Chemistry
- Geoscience
- Physics
- Something not listed here

8. Are there other faculty who specialize in science education in your ... (Mark all that apply)

	Yes	No
Department?		
College?		

9. Which best describes your institution?

- Associate's-degree granting
- Primarily Undergraduate
- Master's-degree granting
- Ph.D.- granting
- Something not listed here

10. Please tell us more about your department and institution.

Full name(s) of home department(s)	
Name of institution	
Location of institution	

2022 Perspectives of Education-focused Science Faculty in the United States

About you and your interests and background...

11. How (if at all) have you been engaged in each of these **Science Education Arenas**?

Science Education Research (e.g., research on issues of student conceptions, teaching and learning strategies, equity and diversity in the sciences, discipline-based science education issues)

K-12 Science Education Activities (e.g., K-12 curriculum development, teacher preparation and professional development projects, diversity and outreach projects)

Undergraduate Science Education Activities (e.g., instructional training for faculty or graduate teaching assistants, formal and informal faculty professional development in science teaching, recruitment/retention outreach projects)

	Science Education Research	K-12 Science Education Activities	Undergraduate Science Education Activities
Published articles in peer-reviewed journals that relate to your scholarly activities	☐	☐	☐
Applied for grants to support your scholarly activities	☐	☐	☐
Presented results of your scholarly activities in this arena at regional, national, or international meetings	☐	☐	☐
Mentored students (undergraduate or graduate)	☐	☐	☐
Mentored colleagues	☐	☐	☐

Something not listed here

[ ]

2022 Perspectives of Education-focused Science Faculty in the United States

About you and your interests and background...

12. Please identify your most advanced formal training in **SCIENCE**

- ◯ Postdoc in science field
- ◯ Ph.D. in science field
- ◯ Master's Degree in science field
- ◯ Bachelor's Degree in science field
- ◯ Minor in science field
- ◯ College level course work in science field
- ◯ Something not listed here

13. Please identify your most advanced formal training in **SCIENCE EDUCATION**

- ◯ Postdoc in science education field
- ◯ Ph.D. in science education field
- ◯ Master's Degree in science education field
- ◯ K-12 Teaching Credential
- ◯ Bachelor's Degree in science education field
- ◯ Minor in science education field
- ◯ College level course work in science education field
- ◯ Something not listed here

14. Consider you current position. To what degree do you agree with the following statements?

	Strongly Agree	Agree	Disagree	Strongly Disagree
I aspire to foster change in how science is taught in my department or institution.	◯	◯	◯	◯
In my opinion I have had success in fostering change in how science is taught in my department or institution.	◯	◯	◯	◯
In my opinion my science education work is **valued** by my peers in my department or institution.	◯	◯	◯	◯
In my opinion my science education work is **understood** by my peers in my department or institution.	◯	◯	◯	◯
In my opinion my science education work is **respected** by my peers in my department or institution.	◯	◯	◯	◯

Please feel free to elaborate if you wish.

15. Consider you current position. How often (if at all) have you experienced negative bias that you believe is likely the result of your professional focus on science education...

	Never	Rarely	Sometimes	Often	Always
by faculty peers within your department or institution?	○	○	○	○	○
by administrators within your department or institution?	○	○	○	○	○
by members of the science community outside of your institution?	○	○	○	○	○

If you have experienced negative bias, please feel free to share your experience(s) here.

About you ...

16. How would you characterize your gender identity?

- ○ Woman
- ○ Man
- ○ Non-binary
- ○ Decline to state
- ○ Something not listed here

17. With which group(s) do you most closely identify?

Please mark all that apply.

- ☐ American Indian or Alaska Native
- ☐ Asian or Asian American
- ☐ Black or African American
- ☐ Hispanic or Latino
- ☐ Something not listed here
- ☐ Native Hawaiian or other Pacific Islander
- ☐ White or Caucasian
- ☐ Decline to state

18. Please consider each prompt about your academic journey.

	Yes	No	Not sure
Are you in the first generation of your family to go to college?	○	○	○
Did you attend a community college as part of your undergraduate degree?	○	○	○

Wrapping up...

19. Please use the space below if there is anything else you'd like to share about your professional experience.

2022 Perspectives of Education-focused Science Faculty in the United States

About your interest in participating in future studies and your colleagues...

If you may be interested in participating in a potential follow up interview study, please copy this link into your browser:
https://www.surveymonkey.com/r/2022FollowUp

If you have education-focused science faculty colleagues who you think should receive this survey, please forward the following survey link to them:
https://www.surveymonkey.com/r/EdFocSciFacUS2022

Thank you!

Thank you for contributing to this investigation of the Perspectives of Education-focused Science Faculty in the United States.

If you would like to learn more about the SFES phenomenon both in the CSU and Nationally please visit: https://tinyurl.com/sfes-pubs.

Thank you for your time.
